# Determinants of Multidrug-Resistant Tuberculosis Clusters, California, USA, 2004–2007

**DOI:** 10.3201/eid1609.100253

**Published:** 2010-09

**Authors:** John Z. Metcalfe, Elizabeth Y. Kim, S.-Y. Grace Lin, Adithya Cattamanchi, Peter Oh, Jennifer Flood, Philip C. Hopewell, Midori Kato-Maeda

**Affiliations:** Author affiliations: University of California, San Francisco, California, USA (J.Z. Metcalfe, E.Y. Kim, A. Cattamanchi, P.C. Hopewell, M. Kato-Maeda);; California Department of Public Health, Richmond, California, USA (S.-Y.G. Lin, P. Oh, J. Flood)

**Keywords:** Bacteria, Mycobacterium tuberculosis, multidrug-resistant tuberculosis, respiratory infections, MDR TB, clusters, molecular epidemiology, isoniazid resistance, phylogeographic lineage, research

## Abstract

Laboratory and epidemiologic evidence suggests that pathogen-specific factors may affect multidrug-resistant (MDR) tuberculosis (TB) transmission and pathogenesis. To identify demographic and clinical characteristics of MDR TB case clustering and to estimate the effect of specific isoniazid resistance–conferring mutations and strain lineage on genotypic clustering, we conducted a population-based cohort study of all MDR TB cases reported in California from January 1, 2004, through December 31, 2007. Of 8,899 incident culture-positive cases for which drug susceptibility information was available, 141 (2%) were MDR. Of 123 (87%) strains with genotype data, 25 (20%) were aggregated in 8 clusters; 113 (92%) of all MDR TB cases and 21 (84%) of clustered MDR TB cases occurred among foreign-born patients. In multivariate analysis, the *katG* S315T mutation (odds ratio 11.2, 95% confidence interval 2.2–∞; p = 0.004), but not strain lineage, was independently associated with case clustering.

## Abstract 2

TOC summary: Type of isoniazid resistance–conferring mutation may be a determinant of genotypic clustering.

In 2007, >500,000 cases of multidrug-resistant (MDR) tuberculosis (TB), defined as resistance to at least isoniazid and rifampin, occurred worldwide (*1*). Although demographic and clinical risk factors for transmission and pathogenesis of both drug-susceptible and drug-resistant *Mycobacterium tuberculosis* have been well described (*2**,**3*), little is known about the microbial factors that influence the generation of secondary MDR TB cases (*4**,**5*).

Community- and population-based molecular epidemiologic studies of isoniazid-monoresistant *M. tuberculosis* (*6**–**8*) have shown that specific resistance-conferring mutations are associated with variable degrees of genotypic clustering, a measure of strain pathogenicity that incorporates host factors, transmissibility of the organism, and capacity of the organism to cause active disease. For example, isoniazid-monoresistant strains with a serine-to-threonine substitution at position 315 (S315T) are more often associated with secondary cases than are strains without the S315T mutation (*6**,**7*), likely because of reduced or absent catalase–peroxidase production (*9*). However, the effects of specific isoniazid resistance–conferring mutations on genotypic clustering in multidrug resistance are less well characterized. The studies reported to date have been limited by inadequate genotypic discrimination (*10**,**11*) and/or nonrepresentative sampling of cases (*10**,**12**–**14*).

California reports the highest annual number of TB cases (*15*), more than one fourth of all MDR TB cases (*16*), and the highest immigration rates in the United States (*17*). We conducted a population-based cohort study of all incident MDR TB cases in California during a 4-year period (January 2004–December 2007) to 1) describe demographic and clinical characteristics of clustering and 2) estimate the effect of specific isoniazid resistance–conferring mutations and strain lineage on genotypic clustering of MDR *M. tuberculosis*.

## Methods

We analyzed culture-positive cases of MDR TB reported to the California TB registry from January 1, 2004, through December 31, 2007. California state law (Health and Safety Code Title 17 §2505) requires reporting of all verified cases of TB, submission of all *M. tuberculosis* isolates to local public health laboratories, and submission of all MDR *M. tuberculosis* isolates to the California Department of Public Health Microbial Diseases Laboratory. Testing for first- and second-line drug susceptibilities was performed at local laboratories or at the Microbial Diseases Laboratory by using BACTEC 460 (Becton Dickinson Diagnostic Instruments, Sparks, MD, USA), MGIT 960 (Becton Dickinson), or the agar proportion method. Some isolates were forwarded to the Centers for Disease Control and Prevention (CDC; Atlanta, GA, USA) for additional second-line drug testing or for confirmation of drug resistance. Patients were included in the study if MDR *M. tuberculosis* was identified on >1 isolate. Demographic and clinical information for all patients with MDR TB was abstracted from state TB surveillance forms (Report of a Verified Case of Tuberculosis), which have high validity (*18*). All protocols were approved by the California Committee for the Protection of Human Subjects and University of California, San Francisco, Committee for the Protection of Human Subjects.

### Characterization of Mutations Associated with Isoniazid and Rifampin Resistance

For each isolate, genomic DNA was extracted from solid media (Lowenstein-Jensen slants, Middlebrook 7H10 or 7H11 agar), liquid media (BACTEC 12 B or MGIT [Becton Dickinson]), or smear-positive sputum sediments. A real-time PCR assay with 6 molecular beacon probes was performed by using an iQ5 iCycler instrument (Bio-Rad, Hercules, CA, USA) to screen for mutations associated with isoniazid and rifampin resistance (*19*). Two molecular beacons that targeted *katG* (codon 311–317) and the *inhA* promoter were used to detect isoniazid resistance–conferring mutations, and 4 molecular beacons that targeted the core region of *rpoB* were used to detect rifampin resistance–conferring mutations. Isolates with mutations in *katG* detected by the wild-type probe were further tested with another molecular beacon that specifically targeted *katG* S315T (AGC–ACC). When molecular beacon analysis did not show *katG* S315T or -c15t *inh*A promoter mutations, the entire *fur*A-*katG* locus (H37Rv: 2153626–2156657, 3,031 bp) was sequenced as described (*6*). Sequence data were generated by using ABI BigDye v3.1 dye terminator sequencing chemistry and the ABI PRISM 3730xl capillary DNA analyzer (Applied Biosystems, Foster City, CA, USA) at the Genomic Core Facility, University of California, San Francisco (www.genomics.ucsf.edu/Sequencing/index.aspx), and were analyzed with ClustalW (www.ebi.ac.uk/Tools/clustalw/index.html).

### Genotyping and Lineage Determination

Spacer oligonucleotide typing (spoligotyping) and mycobacterial interspersed repetitive unit (MIRU) typing were performed in accordance with the Centers for Disease Control and Prevention Universal Genotyping Program procedures (*20*). Spoligotyping was performed by using Luminex-based methods to detect 43 known spacer sequences in the direct repeat locus (*21*). MIRU typing was performed by using the protocol described by Cowan et al. (*22*). A capillary sequencer, CEQ 8000 (Beckman, Fullerton, CA, USA), was used to analyze the number of repeated sequences at each of the 12 loci. IS*6110*-based restriction fragment length polymorphism (RFLP) genotyping was performed following standardized methods (*23*). RFLP patterns were compared by using Bioimage Whole Band Analyzer software version 4.2.1 (Bioimage Corp., Ann Arbor, MI, USA) (*24*). RFLP patterns with <20 identical bands, or >20 bands and differing by no more than a single band, were considered matched. IS*6110* RFLP band assignment was edited by 2 independent readers, and the cluster assignment was confirmed visually.

The phylogeographic lineage of strains was determined from spoligotyping results. Spoligotype families H, LAM, and T, X, S were considered to be of Euro-American lineage; Beijing of East-Asian lineage; EAI of Indo-Oceanic lineage; and CAS of East African-Indian lineage (*5*).

### Definitions

Cases were defined as clustered if >2 isolates from cases reported during the study period shared the same MIRU and spoligotype, matched IS*6110* RFLP, and had specific drug resistance–conferring mutations for isoniazid and rifampin. Clustering was assumed to represent both transmission of *M. tuberculosis* and progression to active disease, leading to secondary case generation within the period of the study. Cases not in a cluster were considered to be the result of reactivation of latent infection. Patients with the earliest case report date within a cluster were regarded as index cases.

### Statistical Analysis

The proportion of clustered MDR TB cases was analyzed as the number of clustered cases divided by the total number of culture-positive cases that occurred during the study period. Because of limited sample size, the independent effects of *katG* S315T and phylogeographic lineage on clustering were estimated by using exact logistic regression methods. Refugee status and sputum smear positivity were included in the model as relevant host risk factors. Refugee resettlement during the study period could bias our results in that overrepresentation of ethnic groups or geographic locations with a high prevalence of particular strain-specific factors (phylogeographic lineage or drug-resistance mutations) could confound the association under study. To examine this influence and potential clustering of TB cases within households or communities related to refugee resettlement, sensitivity analysis was conducted by reestimation of study results after excluding 1) patients immigrating from refugee settings within the past 3 years and 2) the single largest patient cluster, which accounted for 40% of all clustered cases. In a separate analysis, standard logistic regression was used to estimate the effect of *katG* S315T and phylogeographic lineage on sputum smear positivity, again controlling for refugee status.

Odds ratios (ORs) and 95% confidence intervals (CIs) were calculated to measure associations of interest. Categorical data (e.g., sex, foreign vs. US birth, homelessness) were compared by using Fisher exact tests. The Wilcoxon rank-sum test was used to determine differences in the distribution of continuous variables (e.g., age, time from entry into the United States to TB diagnosis). Interaction between the independent variables was assessed separately for each factor by stratification and statistical testing (Breslow-Day with the Tarone correction [*25*] and Zelen test [*26*]), with p<0.2 assumed to indicate the presence of interaction. All p values were 2-sided with α = 0.05 as the significance level. Analyses were performed by using Stata 10 (StataCorp., College Station, TX, USA) and StatExact 8 (CYTEL Software Corp., Cambridge, MA, USA).

## Results

During the study period, 11,395 cases of TB were reported in California, of which 9,037 (79%) had positive cultures. Of these, 8,899 (98%) had isoniazid and rifampin susceptibility results available. Of the 141 (2%) incident MDR TB cases, 123 (87%) had isolates available for MIRU, spoligotyping, and IS*6110* RFLP analysis. Isolates unavailable for genotyping (n = 18) were more often from Los Angeles County; other demographic and clinical characteristics were similar to those of analyzed cases.

Twenty-five MDR *M. tuberculosis* isolates were aggregated in 8 clusters (1 cluster of 10 cases, 1 cluster of 3 cases, and 6 clusters of 2 cases) for an overall cluster proportion of 20% (25/123). Excluding the 8 index cases, 14% (17/123) of all cases were considered to have resulted from recent transmission and rapid progression to disease (secondary cases). Within clusters, a median of 3 months elapsed between successive secondary cases (range 0–20 months). Of the 123 total cases, 113 (92%) occurred among foreign-born patients, with more than half (56%) occurring among immigrants from Mexico, Philippines, the People’s Republic of China, or Vietnam (Table 1). Seven of 8 index cases occurred either in Mexican immigrants (3/8) or recent refugees from Thailand, Lao People’s Democratic Republic, or India (4/8). Median time from patient arrival in the United States to TB diagnosis was approximately twice as long for clustered as for nonclustered cases (4.3 years vs. 2.4 years) and 3 times as long for cases in Mexican-born persons as for cases in persons from other countries (7.3 years vs. 2.3 years).

**Table 1:** Demographic and clinical characteristics of 123 patients with clustered and nonclustered MDR TB infections, California, USA, 2004–2007*

Characteristic	Nonclustered, n = 98†	Clustered, n = 25†	OR‡ (95% CI)	p value
Median age, y (IQR)	39 (30–50)	27 (18–53)	–	0.05
Female gender	47 (48)	11 (44)	0.9 (0.3–2.3)	0.72
Foreign birth	92 (94)	21 (84)	0.3 (0.1–1.8)	0.11
Nation or region of origin				
Philippines	20 (20)	1 (4)	–	<0.001
Mexico	16 (16)	5 (20)		
Vietnam	14 (14)	1 (4)		
China	12 (12)	0		
Central America	6 (6)	0		
India	3 (3)	3 (12)		
Laos	2 (2)	6 (24)		
Thailand	1 (1)	4 (16)		
United States	6 (6)	4 (16)		
All other nations§	18 (18)	1 (4)		
Recent immigration from refugee setting	4 (4)	7 (28)	9.1 (2–45)	<0.001
Median time from US entry to diagnosis, y (IQR)	2.4 (0.2–8.7)	4.2 (0.2–11)	–	0.77
Time from US entry to MDR diagnosis¶				
<3 mo	25 (27)	6 (29)	–	0.42
3 mo–3 y	25 (27)	4 (19)		
>3 y	40 (44)	11 (52)		
Known HIV/TB co-infection	3 (6)	0	–	–
Private healthcare provider	11 (11)	4 (16)	1.3 (0.3–5.4)	0.74
Homelessness	6 (6)	1 (4)	0.7 (0.01–5.9)	1.0
Previous active TB	28 (29)	7 (28)	1.0 (0.3–2.9)	1.0
Sputum-positive AFB smear	60 (66)	16 (70)	1.2 (0.4–3.8)	0.81
Extrapulmonary disease#	7 (7)	1 (4)	0.6 (0.1–3.9)	1.0
Cavitary disease	25 (26)	10 (42)	1.9 (0.7–5.8)	0.22
Median time to culture conversion, mo (IQR)	2.2 (1.3–4.6)	3 (1.4–4.4)		0.57
Median total treatment time, mo (IQR)	25.8 (21.4–28.9)	24.4 (22.6–27.3)		0.56
Treatment failure**	4 (7)	1 (6)	0.9 (0.02–9.7)	0.56
Treatment outcome††				0.70
Completed treatment	70 (83)	17 (81)		
Moved	7 (8)	1 (5)		
Defaulted	2 (2)	0		
Died	5 (6)	3 (14)		

*MDR TB, multidrug-resistant tuberculosis; OR, odds ratios; CI, confidence interval; IQR, interquartile range; AFB, acid-fast bacilli. – indicates OR had no meaning for those specific comparisons. **†Values are no. (%) except as indicated.** ‡ORs describe the association between the characteristic of interest and MDR TB case-clustered status. The denominator for each characteristic excludes missing or unknown values. §**Afghanistan (1), Burma (1), Cambodia (5), Ethiopia (1), Indonesia (1), Mongolia (1), Nepal (1), Peru (2), South Korea (5), and Ukraine (1).** ¶Date of US entry was missing for 2 persons. **#Nonclustered cases: cervical lymph node (5), bone (1), other (1); clustered cases: pleural (1).** **Culture positive after >8 months of treatment; limited to pulmonary TB patients who were alive at diagnosis and had an initial positive sputum culture. ††Treatment outcome available for 105 (85%) cases.

Younger persons were more likely than older persons to harbor strains involved in MDR TB clusters (Table 1). HIV infection was unusual in this patient population; only 3 (4%) of 75 patients with known HIV status were HIV-infected. Twenty-eight percent (35/123) of patients reported a history of active TB; this proportion did not vary between clustered and nonclustered cases (p = 0.75). Eight patients had documented previous treatment in California. Time to culture negativity (2.4 vs. 2.8 months, p = 0.95), treatment failure, or death did not differ between clustered and nonclustered cases (p = 0.57) among 105 (85%) of 123 cases for which data were available.

Seventy-five percent of MDR *M. tuberculosis* strains harbored the isoniazid resistance–conferring mutation *katG* S315T, including all (100%) clustered strains (Table 2). When we controlled for strain lineage and refugee status, *katG* S315T was inversely associated (OR 0.28, 95% CI 0.09–0.89, p = 0.03) with sputum smear positivity (Table 3). Most rifampin resistance–conferring mutations were found between positions 529 and 534 of the *rpoB* gene, likely indicative of the serine to leucine substitution at position 531 (S531L) of the *rpoB* gene (*27*). The association between this mutation and clustering did not reach statistical significance (OR 2.2, 95% CI 0.8–7.4; p = 0.16).

**Table 2:** Isoniazid and rifampin resistance–conferring mutations among 121 clustered and nonclustered MDR TB infections, California, USA, 2004–2007*

Molecular basis for drug resistance	Nonclustered, n = 96, no. (%)	Clustered, n = 25, no. (%)
Isoniazid resistance		
*katG* S315T mutation	66 (69)	25 (100)
Other *katG* mutation†	8 (8)	0
*inhA* promoter‡	23 (26)	0
No *katG* S315T or *inhA* promoter mutation detected§	5 (5)	0
Rifampin resistance¶		
*rpoB* codons 511–518	8 (9)	2 (8)
*rpoB* codons 523–529	25 (27)	4 (16)
*rpoB* codons 529–534	57 (62)	19 (76)
*rpoB* codons 515–521	2 (2)	0

*Two isolates with otherwise complete genotyping data were unavailable for molecular beacon analysis. MDR TB, multidrug-resistant tuberculosis; S315T, serine-to-threonine substitution at position 315. †Novel mutations detected: Y413STOP, T314T (silent), W161G, D61E (Fur A), R145P, P325L, and V633F. ‡**inhA promoter mutation was concomitant with 4/91 (4%) isolates harboring the katG S315T and 2/8 (25%) isolates with katG mutations other than S315T.** §**No mutations detected by molecular beacons; sequencing was not possible for these isolates because of degraded DNA.** ¶Rifampin resistance–conferring mutations were not detected by the molecular beacon assay for 4 isolates.

**Table 3:** Multivariate associations with sputum smear positivity in 102 MDR TB infections, California, USA, 2004–2007*

**Strain**	**OR (95% CI)**	**p value**
**katG S315T**	**0.28 (0.09** **–** **0.89)**	**0.03**
**Euro-American lineage†**	**1.0**	–
**East-Asian lineage**	**0.31 (0.11** **–** **0.88)**	**0.03**
**Indo-Oceanic lineage**	**0.22 (0.06** **–** **0.86)**	**0.03**
**Refugee status**	**2.02 (0.43** **–** **9.45)**	**0.37**

*MDR TB, multidrug-resistant tuberculosis; S315T, serine-to-threonine substitution at position 315; OR, odds ratio; CI, confidence interval. **†Reference.**

MDR *M. tuberculosis* isolates were distributed among East-Asian (47%), Euro-American (30%), Indo-Oceanic (20%), and East African–Indian (3%) phylogeographic lineages. Lineage could not be established for 11 cases. On univariate analysis, East-Asian strain lineage was associated with clustering (Table 4), but not with adverse outcome (death or treatment failure). Indo-Oceanic strains produced no secondary cases.

**Table 4:** Univariate associations of phylogeographic lineage with 112 clustered and nonclustered MDR TB infections, California, USA, 2004–2007*

Strain lineage	Nonclustered, n = 87, no. (%)	Clustered, n = 25, no. (%)	OR (95% CI)	p value
East-Asian	37 (43)	17 (68)	2.87 (1.03–8.48)	0.04
Euro-American	26 (30)	8 (32)	1.10 (0.36–3.12)	0.81
Indo-Oceanic	21 (24)	0	–	0.003
East African–Indian	3 (4)	0	–	1.0

*N = 112. Lineage could not be established by spoligotyping for 11 (8.9%) cases. MDR, multidrug-resistant tuberculosis; OR, odds ratio; CI, confidence interval.

Clustering was independently associated with *katG* S315T (OR 11.2, 95% CI 2.2-∞; p = 0.004) and refugee status (OR 6.0, 95% CI 1.2–36.2; p = 0.03) in exact multivariate analyses in which strain lineage and sputum smear positivity were controlled for (Table 5). The estimated association between *katG* S315T and case clustering did not appreciably change in sensitivity analyses that excluded either all recently arrived refugees or the single largest patient cluster.

**Table 5:** Multivariate exact logistic regression for associations with clustering among **MDR TB** infections, California, USA, 2004–2007*

**Strain**	**OR (95% CI)**	**p value**
**katG S315T**	**11.2 (2.2** **– ∞** **)**	**0.004**
**East-Asian lineage**	**3.1 (0.9** **–** **12.2)**	**0.08**
**Sputum smear–positivity**	**3.1 (0.9** **–** **11.7)**	**0.07**
**Refugee status**	**6.0 (1.2** **–** **36.2)**	**0.03**

*MDR TB, multidrug-resistant tuberculosis; OR, odds ratio; CI, confidence interval.

## Discussion

In this 4-year population-based molecular epidemiologic study, transmission followed by secondary case generation contributed to ≈14%, or 1 of every 7, MDR TB cases in California. Clustered cases occurred more often among younger persons and persons who had emigrated from Mexico and refugee settings in Southeast Asia. In addition, pathogen-specific factors were associated with clustering of MDR TB cases, independent of traditional clinical and demographic risk factors.

The proportion of MDR TB cases attributed to transmission in California was higher than that reported by other investigators in most low incidence settings (*28**–**32*). The largest clusters in our study resulted from MDR TB outbreaks in California after resettlement of Hmong refugees in 2005–2006 (*33*) and resettlement of Tibetan refugees in 2001–2006. The associations between pathogen-specific factors and case clustering could be due to regional differences in strain prevalence and preferential migration of persons with specific strains to California. We attempted to control for these factors by using highly stringent criteria to define clustered cases and by adjusting for refugee status in our multivariate model. However, without detailed contact information and contact tracing, we cannot be certain of the extent to which transmission or progression to active disease occurred within or outside California. Given that most clustered cases occurred among persons residing in the United States for >3 years and that US-born persons were involved in 3 of 8 clusters, at least some proportion of MDR TB transmission is likely to have occurred in California. This observation suggests that although most MDR TB cases in the United States are related to the migration of persons already infected with drug-resistant *M. tuberculosis*, a small but notable proportion may be due to ongoing transmission.

Heterogeneity in the reproductive success of drug-resistant *M. tuberculosis* is now well established (*34**,**35*). In this study, the *katG* S315T mutation was the only isoniazid resistance–conferring mutation found among clustered MDR TB cases. The high prevalence of *katG* S315T among MDR strains (*12**,**14**,**36**–**38*) and the association of this mutation with increased secondary case generation among isoniazid-monoresistant strains (*6**,**7*) have been documented. We report that the *katG* S315T isoniazid resistance–conferring mutation retains an independent effect on clustering of MDR TB cases despite the presence of mutations that confer resistance to additional drugs. In particular, secondary case generation did not significantly vary according to site of *rpoB* mutations that confer rifampin resistance, which supports the hypothesis that these are no-cost mutations or that compensatory mutations commonly exist (*39*).

The *katG* S315T mutation is thought to preserve fitness through the relative preservation of catalase-peroxidase production (*9*), although whether this mutation is associated with different clinical phenotypes is unknown. In this study, we noted an inverse association between presence of the *katG* S315T mutation and sputum smear positivity. In addition, the *katG* S315T mutation was associated with case clustering, independent of sputum smear status. These findings suggest that the *katG* S315T mutation may preserve the ability of *M. tuberculosis* to transmit and cause secondary cases through mechanisms unrelated to conventional indices of disease severity, such as the presence of abundant acid-fast bacilli in sputum.

Our findings are clinically useful for at least 2 reasons. First, MDR TB in California is occurring predominantly among patients who were not born in the United States, with some cases from recent transmission and rapid progression to disease. Our study suggests that in California, younger persons and persons who have emigrated from Mexico and from refugee settings may be at higher risk for transmitting MDR *M. tuberculosis*. Likewise, our findings reinforce the need for giving priority to screening and prevention activities in immigrant communities and US investment in international TB control (*40*). Second, if our results are verified in other settings, TB-control programs should consider pathogen-specific factors such as isoniazid resistance–conferring mutations when planning the intensity of contact investigation and secondary case-finding activities.

This study has several limitations. First, our estimates of case clustering are imprecise because of the limited number of MDR TB cases observed during the study period. However, these estimates are the best currently available, given that the data make up the largest population-based TB registry in the United States. Second, although our definition of genotypic clustering was highly rigorous, the lack of detailed epidemiologic information precluded confirmation of transmission within California. Third, because our study did not include pan-susceptible or isoniazid-monoresistant strains, we cannot comment directly on MDR *M. tuberculosis* pathogenicity relative to these groups. Lastly, our analyses implicitly assume independence of outcome events, and household or community-level factors potentially associated with clustering were not available. Future studies should be designed so that statistical methods can be used that are able to accommodate the possible effects of within-household clustering.

We found a substantial proportion of MDR TB cases and case clustering in California among non–US-born persons, and the *katG* S315T mutation was independently associated with clustering. Validation of these findings in larger cohorts and in different population settings may have crucial public health consequences.
